# Nutrition-Related Content on Instagram in the United States of America: Analytical Cross-Sectional Study

**DOI:** 10.3390/foods11020239

**Published:** 2022-01-17

**Authors:** Hector José Tricas-Vidal, María Concepción Vidal-Peracho, María Orosia Lucha-López, César Hidalgo-García, Ana Carmen Lucha-López, Sofía Monti-Ballano, Jaime Corral-de Toro, Sergio Márquez-Gonzalvo, José Miguel Tricás-Moreno

**Affiliations:** 1 Unidad de Investigación en Fisioterapia, Universidad de Zaragoza, Domingo Miral, s/n, 50009 Zaragoza, Spain; tricasdpt@gmail.com (H.J.T.-V.); cvidal@unizar.es (M.C.V.-P.); analucha@unizar.es (A.C.L.-L.); smonti1395@gmail.com (S.M.-B.); jaimecorral.fisio@gmail.com (J.C.-d.T.); serguomg@gmail.com (S.M.-G.); jmtricas@unizar.es (J.M.T.-M.); 2 School of Health Professions, University of Mary Hardin Baylor, 900 College St., Belton, TX 76513, USA; 3 Department of Endocrinology and Nutrition, Hospital Royo Villanova, SALUD, Barrio San Gregorio, s/n, 50015 Zaragoza, Spain

**Keywords:** social media, diet, food and nutrition, profile user, evidence-based practice

## Abstract

Background: The Internet is today the largest platform for food distribution, and there are concerns about the impact that digital marketing has in the field of nutrition by promoting non-evidence-based recommendations. The purpose of this study was to describe the user profile that draws on Instagram to follow nutrition-related content versus not, and to analyze the frequency and type of content of the information provided by nutritional influencers. Methods: A cross-sectional study involving randomly selected United States residents having an Instagram account was performed. Participants completed an anonymous online survey link. Results: From 898 respondents, 78.7% were women, and 75.6% were Millennials. Scientific evidence present in the posts was 14.3%. Influencers promoted a product or a brand in more than 90% of posts. Women followed more nutrition-related content than men (*p* < 0.001). Millennials, followed by Generation-Z, followed more nutrition-related content (*p* < 0.001). There were no significant relationships between the following status of nutrition-related content with BMI, type of job, household income, education degree, or smoking habits. Conclusions: Women and Millennials followed more nutrition-related content. Scientific evidence was scarce and commercial interest in the network was evident. The vast majority of the posts were not based on scientific evidence and instead promoted a product/supplement.

## 1. Introduction

Social networks are the most revolutionary phenomenon in communication after television [1]. They allow increasing social interaction on the Internet and the intention to buy [2]. In a comprehensive review of the health implications of image-based networks, it has been concluded that their role in health is unknown [3]. The great advantage of the networks is that they are free, and the disadvantage is their financial motivations.

Therefore, the Internet is now the largest platform for the food market, and there is concern that digital marketing, in the field of nutrition, does not have recommended effects, similar to television [1]. However, for the scientific community, networks’ interest is based on access to training or images and dialogue and interconnection with open groups, not only of experts [1]. In fact, governments have published guides to structure communication in social media and scientific nutritional activity [4]. It is known that platforms can help spread messages, promote behavior changes, and structure educational, nutritional programs online, particularly in younger people [5]. In addition, the resources that a system has in the prevention of chronic diseases are associated with greater use of Instagram and health information, with better results in health protection behaviors, particularly among university students [6].

Since its launch in 2010, as an online smartphone app platform that enables users to share videos and photos with their followers and in other networks, Instagram has increased its growth and profitability, particularly after the acquisition by Facebook in 2012. Instagram is an image-based social media platform that promotes a new form of communication and self-expression based on images and photos, mainly showing thin, muscular, and unrealistic body ideals [7]. The Pew Review Center surveyed the Social Media Use in 2018 among Americans. Results showed that Instagram was used by 35% of US adults. From the previous survey in 2016, there is an increment in user usage of seven percentage points from the 28% reported in 2016. Out of those Americans who used Instagram, 60% of users indicated that they visit these platforms daily. In 2016, 51% of Instagram users were daily visitors [8]. The number of followers is increasing so rapidly on Instagram that represents an ideal tool for electronic word of mouth [9]. The audience is such that in the United Kingdom, 50% of children aged 8–11 use Instagram [10,11].

Instagrammers are social media personalities, or “Influencers” on Instagram [12]. There are numerous nutritional Instagrammers, who are any people that provide nutritional information on Instagram with or without being a certified nutritionist or registered dietitian.

The impact that a platform of this type may have on the nutritional behavior and health habits of users led us to the design of the study with two main objectives: to describe the user profile that draws on Instagram to follow nutrition-related content versus not, and to analyze the frequency and type of content of the information provided by nutritional influencers.

## 2. Materials and Methods

### 2.1. Study Design

An analytical cross-sectional study involving US citizens and residents recruited via email to participate in an anonymous online survey.

### 2.2. Setting

An invitation via email account with the survey link was sent to current or graduated students from Queens University of Charlotte, The University of Kentucky, Oakland University, and the University of Mary Hardin Baylor. Moreover, the survey link was published on Facebook. Diffusion of the link was carried out with a snowball effect. The survey link was sent to participants via Survey Monkey platform, and responses were collected for later analysis. Thus, a convenient sampling technique was performed.

### 2.3. Participants

To calculate the sample size, the US Population was used: 329,256,465 (July 2018 statistics) according to CIA World Factbook. The expected proportion used was 35% because Instagram was used in 2018 by 35% of American adults, according to Smith and Anderson [8]. The sample size was calculated using the GRANMO calculator [13], with the selection of population estimation, confidence level 0.95 with a desired precision with the selected confidence level of +/−3.5 percent units. A minimal number of 226 randomly selected was obtained.

To achieve better representativeness of the sample, researchers ultimately surveyed 898 participants that used Instagram. The inclusion criteria were that survey participants must be over 18 years old. The exclusion criteria were that they needed to have an Instagram account. None of the participants were compensated for participating in this research.

### 2.4. Ethics and Human Subject Protection

The Academic Commission of the Doctoral Program in Health and Sports Sciences of the University of Zaragoza approved the study (protocol code 496/29 August 2019), which complied with the ethical requirements of the Declaration of Helsinki [14].

The study did not ask participants questions regarding, race, sexual identity, religion, political views, or other questions that could break the law regarding research ethics. After clicking on the link to be directed to the survey, all subjects have read the following agreement before starting the survey.

The statement said: “With your participation in this study you will help us understand how you attempt to use Instagram. This survey is anonymous, so we do not collect identifying information such as your name, email, or IP address. By clicking NEXT, you will consent to the University of Zaragoza and the College of Health and Sport Sciences to use the information provided in this study. Information will be destroyed after the Study is completed”.

By clicking the bottom, “I have read through the consent, and I agree to participate in this survey”, the subject provided consent.

### 2.5. Data Sources

#### 2.5.1. Socio-Demographic Characteristics of the Sample

Participants were asked to provide the following information on its socio-demographic characteristics.

- State of residence.
- Gender. Male/female/others.
- Age. Age was categorized according to the next generations: generation-Z (born 1997–2012); millennials (born 1981–1996); generation-X (born 1965–1980); boomers (born 1946–1964) [15].
- Height in feet and inches and weight in pounds. BMI was calculated with these data. BMI = 703 × weight (pounds)/[height (inches)]^2^.

BMI was categorized according to US Centers for Disease Control and Prevention BMI guidelines. For BMI under 15 is considered severely underweight, between 16 and 18.4 is considered underweight, between 18.5 and 24.9 is considered normal, between 25 and 29.9 is considered overweight, above 30 is considered obese.

- Type of job. The next categories were possible: Unable to work; Self-Employed; Retired; Student; Unemployed (Not currently looking for a job); Unemployed (Currently looking for a job); Employed Part-time (Less than 40 h per week); Employed Full-time (40+ hours a week).
- Sample household annual income. It was classified in: Below USD 10 K; USD 10–50 K; USD 50–100 K; USD 100–150 K; over USD 150 K; K = $1000.
- Degree earned. Participants were asked about their highest education degree accomplished. Doctorate Degree; Master’s Degree; Bachelor’s Degree; Associate Degree; Trade/Technical/Vocational training; some college credit, no degree; high-school graduate or equivalent.
- Smoking habits. Yes/No/Occasionally.
- How long the participants have regularly consulted Instagram (months).
- Hours per week on Instagram checking for nutrition or exercise.

#### 2.5.2. Instagram Attitudes

Participants were asked to answer the following questions about their Instagram use habits.

- Follow a Nutritional Influencer on Instagram. No/Yes. Name some of your favorite ones: (Open Question).
- Follow any of the advice, recipes, or meals plans from nutrition influencers on Instagram. No/Yes. This question was renamed as the variable “follow status of nutrition-related content”.
- Ever look up influencer’s accreditations to confirm they are a registered dietician or certified nutrition specialist. No/Yes.
- Ever check the accuracy of the advice, recipes, or meal plans posted by those influencers. No/Yes.
- Ever hire an online personal trainer. No/Yes.

#### 2.5.3. Impact on Body Image Due to Following Fitness or Nutrition Instagrammers

Participants were asked to describe the impact on their body image, produced by influencers, in one of the following three categories: positive, negative, and neither.

#### 2.5.4. Physical Activity

Physical activity carried out by the participants was registered with the International Physical Activity Questionnaire (IPAQ) [16]. Data were recoded.

- Recodification of variables for vigorous exercises: less than 75 min per week (1) and more than 75 min per week (2). (1) = do not meet the 75 min/week recommended guidelines in for substantial health benefit; (2) = do meet the 75 min/week of vigorous week recommended in guidelines for substantial health benefit.
- Recodification of variables for moderate exercises: less than 150 min per week (1) and more than 150 min per week (2). (1) = do not meet the 150 min/week of moderate exercise recommended in guidelines for substantial health benefit; (2) = do meet the 150 min/week of moderate week recommended in guidelines for substantial health benefit.
- Recodification of variables for time spent seating: low risk indicates sitting less than 4 h per day; medium risk indicates sitting 4 to 8 h per day; high risk indicates sitting 8 to 11 h per day; very high risk indicates sitting more than 11 h per day [17].

### 2.6. Statistical Analysis

SPSS v25 was used to compute frequency and descriptive statistics. To examine the relationship between variables, “follow status of nutrition-related content” was established as independent variable. If the dependent variable was qualitative Chi-square was used and if it was quantitative U-Mann–Whitney test was used.

Afterwards, a generalized linear model was conducted with “follow status of nutrition-related content” as the dependent variable, and with the variables with significative relationships previously detected as predictors. The model applied was binary logistic. The model effects were main effects, creating a main-effects term for each selected variable. The parameter estimation method was hybrid, and the scale parameters were fixed value.

From the open question “name some of your favorite nutritional influencers on Instagram”, researchers analyzed the different posts made by the named influencers according to the following categories:

- Scientific evidence on the post: the post required reference or citation to primary research to consider that scientific evidence was present.
- Post promoted a product or supplement: the post contained any reference to a nutritional product or supplement.
- Post promoted a brand: the post contained any reference to a type of nutritional product manufactured by a particular company under a specific name.
- Post about a recipe: the post contained a set of instructions for preparing a particular dish, including a list of the ingredients required and the nutritional information of the recipe (calories and nutritional composition).
- Post suggested to the follower what to eat: the post suggested eating one food or group of foods to obtain a health benefit.

Once the categories extracted from the posts were identified, they were analyzed with descriptive statistics.

## 3. Results

### 3.1. Sample Description

#### 3.1.1. Socio-Demographic Characteristics of the Sample

Out of 898 participants, 49 states were represented, no participants were from the state of West Virginia (Table S1).

Out of 896 participants in this observational study, 20.6% (*n* = 185) were male, 78.7% (*n* = 705) were female, and 0.7% (*n* = 6) identify themselves as other (Table 1).

All generations were represented in this study (Table 1). Out of 898 participants, 75.6% (*n* = 679) belong to the Millennial generation. This group of participants was between 23 and 38 years old when the survey was taken (August 2019–December 2019); 11.5% (*n* = 103) belong to Generation Z (between 18 and 22 years); 11.4% (*n* = 102) belong to Generation X, (39 and 54 years old); and 1.6% (*n* = 14) belong to the Baby Boomer generation (55 and 73 years old when the survey was administered).

Out of all participants, 0.7% (*n* = 6) were severely underweight, 1.7% (*n* = 15) were considered underweight, 57.6% (*n* = 516 participants) were considered to have a normal BMI, 25.7% (*n* = 234) were considered overweight, and 14.3% (*n* = 127) were considered obese (Table 1).

Out of 898 participants (Table 1), 53.9% were employed full time (40+ h a week), 28.7% were students, 9.3% were employed part-time (less than 40 h per week), 3.5% of participants reported to be self-employed, 2.2% reported to be unemployed looking for a job, 1.6% reported to be unemployed not looking for a job, 0.5% were unable to work, and 0.3% were retired.

The household income reported by the participants was (K = $1000): 37.1% reported to earn between USD 50 and 100 K, 21.2% reported to earn between USD 10 and 50 K, 15.4% reported to earn between USD 100 and 150 K, 13.3% reported to earn over USD 150 K, and 13.0% reported to earn less than USD 10 K per year (Table 1).

Participants were asked about their highest education degree accomplished: 43.6% of the participants reported to have completed their bachelor’s degree; 28.3% have completed their doctorate degree; 14.9% have completed their master’s degree; 5.8% reported the completion of some college credits, but without completing the degree requirements; 3.6% reported to have their high school diploma or GED (General Education Development) equivalent; 3.2% reported to have an associated degree; and 0.6% reported to have trade, technical, or vocational training (Table 1).

Participants were asked about their smoking habits, 93.4% (*n* = 839) reported not smoking, 4.2% (*n* = 38) reported smoking occasionally or socially, and 2.3% (*n* = 21) reported to smoke (Table 1).

The participants (*n* = 794) have consulted for nutrition or exercise advice/information on Instagram a mean of 23.6 months (SD = 24.1) (Table 1).

#### 3.1.2. Instagram Attitudes

Out of 898 participants, 56.2% (*n* = 505) said they do not follow any nutrition influencers on Instagram; 43.8% (*n* = 393) of participants said they do follow nutrition influencers on Instagram (Table 1).

Out of 898 participants, 67.8% (*n* = 609) said they have never followed nutrition-related content on Instagram; 32.2% (*n* = 289) of participants said they followed nutrition-related content.

Out of 898 participants, 77.6% (*n* = 697) said they have not checked the nutrition Instagram influencers’ accreditation; 22.4% (*n* = 201) of participants said they checked the nutrition Instagram influencers’ accreditation (Table 1).

Out of 898 participants, 73.9% (*n* = 664) said they have not checked the nutritional evidence of the recipes from the Instagram influencers; 26.1% (*n* = 234) of participants said they have checked the nutritional evidence of the recipes from the Instagram influencers (Table 1).

Out of 898 participants, 81.5% (*n* = 732) said they have not considered or ever hired personal training services on Instagram from a fitness influencer; 18.5% (*n* = 166) said they have considered or have hired before personal training services on Instagram from a fitness influencer (Table 1).

#### 3.1.3. Impact on Body Image Due to Following Fitness or Nutrition Instagrammers

Out of 898 participants, 54% (*n* = 481) participants claimed neither impact on their body image by following Instagrammers; 30% (*n* = 268) participants claimed a positive impact on their body image by following Instagrammers; 16% (*n* = 149) participants claimed a negative impact on their body image by following Instagrammers (Table 1).

#### 3.1.4. Physical Activity IPAQ Questionnaire

Of the total sample, 770 subjects performed vigorous physical activity and 751 moderate physical activity; 13.9% performed vigorous physical activity for less than 75 min a week, and 86.1% more than 75 min a week; 47.3% performed moderate physical activity for less than 150 min a week; and 52.7% more than 150 min a week. Regarding the time they sat down and its relationship with cardiovascular risk, 35.1% were at low risk, 45.8% at medium risk, 9.9% at high risk, and 9.2% at very high risk (Table 1).

### 3.2. Relation Analysis between Variables

#### 3.2.1. Relations between Socio-Demographic Characteristics of the Sample and the Following Status of Nutrition-Related Content

Women followed more nutrition-related content than men (*p* < 0.001). Millennials, and Generation-z, followed nutrition-related content on Instagram more than Generation-X or Boomers (*p* < 0.001) (Table 1).

There were no significant relationships between following any advice, recipes, meal plans from nutrition influencers on Instagram with BMI, type of job, household income, highest education degree earned, or smoking habits (Table 1).

There was a relationship between how long consulting Instagram and hours per week on Instagram checking for nutrition or exercise with the following status of nutrition-related content (*p* < 0.001) (Table 1).

#### 3.2.2. Relations between Instagram Attitudes of the Sample and the Following Status of Nutrition-Related Content

There was a significant difference between groups in the follow status of nutrition-related content on Instagram related to following nutrition influencers (*p* < 0.001). Among those who followed nutrition-related content on Instagram, 81.3% followed a nutritional influencer (Table 1).

Among the people who followed nutrition-related content on Instagram, 43.6% inquired about the influencer’s academic accreditation (*p* < 0.001), and 54.3% inquired about the accuracy of the information (*p* < 0.001). Among people who followed nutrition-related content on Instagram, 26.3% considered hiring or had hired a personal trainer, compared to 14.8% of those who did not follow nutrition-related content (*p* < 0.001) (Table 1).

#### 3.2.3. Relations between Instagram Body Image Influenced by Fitness and Nutrition Influencers and the Follow Status of Nutrition-Related Content

There were 898 valid responses. There is a significant difference between groups in the following status of nutrition-related content on Instagram in relationship with influence on body image (*p* < 0.001) (Table 1).

Among those who followed nutrition-related content on Instagram, 48.4% reported positive reinforcement in their body image, 17.6% reported negative reinforcement, and 33.9% reported no influence (Table 1).

#### 3.2.4. Relations between Physical Activity and the Follow Status of Nutrition-Related Content

There were no significant relationships between the following status of nutrition-related content on Instagram and vigorous physical activity, moderate physical activity, and hours of sitting Table 1).

#### 3.2.5. Generalized Linear Model

The generalized linear model with the variable, “follow status of nutrition-related content” as the dependent variable only detected main effects for the variables:

- Follow a Nutritional Influencer on Instagram (*p* < 0.001).
- Ever check the accuracy of the advice, recipes, or meal plans posted by those influencers (*p* < 0.001).
- Hours per week on Instagram checking for nutrition or exercise (*p* < 0.002).
- Impact on body image due to following Instagrammers (*p* < 0.031).

### 3.3. Posts by the Named Nutritional Influencers Analysis

#### 3.3.1. Category 1: Scientific Evidence on Post

It was found that 85.7% (*n* = 180) of posts are not based on scientific evidence; 14.3% (*n* = 30) of posts are based on scientific evidence (Table 2).

#### 3.3.2. Category 2: Post Promoted a Product or Supplement

From 210 posts, 91.4% (*n* = 192) of posts promote a product or supplement, while 8.6% (*n* = 18) do not promote a product or supplement (Table 2).

#### 3.3.3. Category 3: Post Promotes a Brand

From 210 posts, 93.8% (*n* = 197) of posts promote a brand, 6.2% (*n* = 13) do not promote any specific brands (Table 2).

#### 3.3.4. Category 4: Post about a Recipe

From 210 post, 65.7% (*n* = 138) of posts do not post content about recipes, 34.3% (*n* = 72) do post recipe content on their Instagram (Table 2).

#### 3.3.5. Category 5: Post Suggest Follower What to Eat

We found that 86.7% (*n* = 182) of posts suggest to followers what to eat, 13.3% (*n* = 28) do not suggest what to eat (Table 2).

## 4. Discussion

### 4.1. Principal Results

From 898 respondents throughout the US, all the states except West Virginia were represented. A total of 78.7% were women, a higher percentage than in the Instagram global statistics 2019 (51%) [18]; although, it coincides with other studies on the influence of healthy food posts on purchase intention (*n* = 200), where 78.2% were also women [9]. However, our study agreed with Instagram global statistics 2019 that the largest age group in our population were the Millennials: 75.6% versus 71% in Instagram global statistics 2019 [18]. A higher percentage of women and millennials followed Instagram for nutrition-related content. The topic of food trendy on Instagram, and 69% of millennials take a photo or video of their food before eating. Food fanatics connect to Instagram an average of 18 times a day [18]. Studies are showing that Instagram promotes orthorexia nervosa, or obsession with healthy eating, which does not appear with other platforms, and, in the literature, this behavior occurred in more than 49% of Instagram users (general population < 1%) [19]. It has been shown that women who quit Instagram for only a week reported significantly higher life satisfaction and positive affect levels than women who kept using it [20].

In our group, although 57.6% were normal-nourished according to BMI, 25.7% were overweight, and 14.3% were obese; that is, 40% showed weight alterations, measured by BMI. BMI plays an essential role concerning to healthy food posts on Instagram, and in people with obesity, it increases their motivation and limits depressive attitudes. In general, women are more inspired by images that lead them to be thinner or more muscular [9]. However, as shown before, we did not find a relation between the following status of nutrition-related content from Instagram nutrition influencers and BMI in our population [21].

In 2018, the percentage of people 25 years and older in the United States of America who had completed a bachelor’s degree or higher was 35.0 percent [22]. In this study, 86.8% of the sample had completed a bachelor’s degree or higher.

We did not find relationships between the following status of nutrition-related content with the type of job, household income, smoking habit, or education degree.

It was found that 43.8% of the sample followed an influencer in nutrition; 22.4% considered the accreditation of the influencer in nutrition; 26.1% looked at the safety of recipes; and 18.5% considered hiring a personal trainer. We also found that 43.6% of the participants who followed nutrition-related content considered the accreditation of the influencer, and 54.3% looked at the safety of recipes. The high education status of the sample may have favored that around half of the subjects following nutrition-related content, checked the accreditation of the influencer and the safety of the recipes.

On the one hand, our results reflect the power of the social network and, on the other, the lack of security about food content that is more related to commerce than science. In fact, in some studies, 49% of consumers learn about food through social networks. Word of mouth seems to play a fundamental role in the consumer [9]. Instagram inspires many users, allowing them to escape their own lives and become hooked on what other users are doing and connect with visual culture. The influencers define the diet, the type of exercise, the supplements to consume, the brand of sports products, and promise that their combination leads to happiness. This creates a dependency relationship between the influencer and his followers, which will be evaluated in younger populations [23].

In fact, in our study, the scientific evidence of the posts was only 14.3%, which generates misinformation [1]. Moreover, the commercial interest of the network [18] was manifested. In our group, the posts promoted a product or a brand in more than 90% of the cases, and the posts suggested to the followers what they should eat in more than 86%. These results coincide with the fact that social networks increase consumer confidence, the intention of buying, and the popularity of brands [2].

These results allow us to reflect on the need to maintain free access to the network but also to control the content in relation to health, knowing its scientific origin and qualification of the signatory. This fact is something that Instagram will have to consider, as other platforms have done, due to responsibility, with fake news [1]. Therefore, social networks increase business opportunities more attractively [24] because consumers are more interested in the recommendations of other users than in the information of the seller. The perceived usefulness would be one of the main pillars of this technological success [25]. Therefore, interactions between consumers increase their level of trust and decrease the perceived risk, facilitating consumption [2]. In general, Instagram likes are more linked to posts with moderate calorie intake than very high- or low-calorie intake [26]. When healthy products are advertised, the user’s physical appearance seems to have the greatest effect on the consumer’s intention to buy instead of gender and popularity [9].

The results in our study show that only 30% had a positive impact on their body image, 16% negative; although, the effect on half of the sample was neutral. However, among the participants who followed nutrition-related content on Instagram, 48.4% reported a positive impact, 17.6% a negative impact, and 33.9% reported a neutral impact. On the other hand, it has been established that exposure to attractive celebrities from Instagram can be detrimental to women’s body image [27]. However, due to the characteristics of our sample, degree of education, or work status, not much influence on body image was observed in general.

We found that 86.1% of the population performed vigorous physical activity for more than 75 min per week, and 52.7% performed moderate physical activity for more than 150 min per week. Regarding the time they remained seated and its relationship with cardiovascular risk, people with a medium risk was 45.8%. The data showed the high physical activity status of the sample, considering that among United States of America adults, in 2016, only 26% of men and 19% of women reported performing sufficient physical activity [28].

However, we did not find any relations between the following status of nutrition-related content and intense, moderate physical activity or hours of sitting. Our population, with a significant percentage that performed physical activity, could reflect the relationship between interest in food and healthy life, given the frequent association between both topics [18].

Despite the multiple associations found for the following status of nutrition-related content variable, only four emerged from the generalized linear model analysis, which showed that the user profile that followed nutrition-related content on Instagram: followed more, a nutritional influencer; checked more, the accuracy of the advice; and stayed more hours per week on Instagram checking for nutrition or exercise. People whose body image was impacted positively due to following fitness or nutrition influencers had more reasons to follow nutrition-related content on Instagram.

### 4.2. Limitations

This study is subject to some limitations. It is a study that relies on self-reports and is prone to response bias. BMI calculations are subject to possible bias, as height and weight were provided by participants. The questions in relation to Instagram attitudes may have included more options to answer than yes/no, such as rarely, sometimes, and frequently to learn more about these trends, but the need to simplify the survey to facilitate its completion prevailed. Generalizability of research findings might be limited because the sample, due to size, may be not representative of the 35% of the American adults that use Instagram. Furthermore, the sample was not stratified according to the states, so the representation is not fair for all of them. Due to the snowball effect of sending the survey to former students, the population has a high degree of education in comparison with the current percentage of the US population [18].

## 5. Conclusions

Women and Millennials followed more nutrition-related content, considering that this is the generation predominantly represented in the sample. User profiles that followed nutrition-related content on Instagram followed a nutritional influencer, checked the accuracy of the advice, stayed more hours per week on Instagram checking for nutrition or exercise, and had a positive impact on their overall body image due to following fitness or nutrition influencers. Scientific evidence was scarce and commercial interest in the network was evident. The vast majority of influencers’ posts were not based on scientific evidence and instead promoted a product/supplement.

## Figures and Tables

**Table 1:** Characteristics of the sample and relations with the follow status of nutrition-related content on Instagram.

Characteristics			Follow Status of Nutrition-Related Content		
			No	Yes	*p*-Value
**Gender (*n* = 896)**		**%**	**%**	**%**	
	Male (*n* = 185)	20.6	24.2	13.2	<0.001 ^a^
	Female (*n* = 705)	78.7	74.8	86.8	
	Other (*n* = 6)	0.7	1.0	0.0	
**Generation (*n* = 898)**		**%**	**%**	**%**	
	Generation-z (born 1997–2012) (*n* = 103)	11.5	12.0	10.4	<0.001 ^a^
	Millennials (born 1981–1996) (*n* = 679)	75.6	72.7	81.7	
	Generation-x (born 1965–1980) (*n* = 102)	11.4	13.0	8.0	
	Boomers (born 1946–1964) (*n* = 14)	1.6	2.3	0.0	
**Body Mass Index (*n* = 898)**		**%**	**%**	**%**	
	Severely underweight (<15) (*n* = 6)	0.7	0.8	0.3	0.544
	Underweight (16–18.4) (*n* = 15)	1.7	1.3	2.4	
	Normal (18.5–24.9) (*n* = 516)	57.6	56.5	59.5	
	Overweight (25–29.9) (*n* = 234)	25.7	26.8	24.6	
	Obese ≥ 30 (*n* = 127)	14.3	14.6	13.1	
**Type of job (*n* = 898)**		**%**	**%**	**%**	
	Unable to work (*n* = 4)	0.5	0.5	0.3	0.460
	Self-employed (*n* = 33)	3.5	3.6	3.8	
	Retired (*n* = 3)	0.3	0.5	0.0	
	Student (*n* = 255)	28.7	27.3	30.8	
	Unemployed (no looking job) (*n* = 15)	1.6	1.5	2.1	
	Unemployed (looking job) (*n* = 19)	2.2	1.6	3.1	
	Employed Part-time (less 40 h) (*n* = 82)	9.3	9.0	9.3	
	Employed Full-time (40 h) (*n* = 487)	53.9	56.0	50.5	
**Household income (*n* = 898)**		**%**	**%**	**%**	
	Below USD 10 K (*n* = 114)	13.0	13.0	13.1	0.774
	USD 10–50 K (*n* = 185)	21.2	20.4	23.2	
	USD 50–100 K (*n* = 326)	37.1	38.3	34.6	
	USD 100–150 K (*n* = 137)	15.4	15.6	14.9	
	Over USD 150 K (*n* = 117)	13.3	12.8	14.2	
**Highest degree earned (*n* = 898)**		**%**	**%**	**%**	
	Doctorate Degree (*n* = 252)	28.3	29.1	26	0.120
	Master’s Degree (*n* = 136)	14.9	15.1	15.2	
	Bachelor’s Degree (*n* = 388)	43.6	41.4	47.1	
	Associate Degree (*n* = 28)	3.2	3.4	2.8	
	Trade/Technical/Vocational training (*n* = 5)	0.6	0.8	0.0	
	Some college credit, no degree (*n* = 55)	5.8	5.7	6.9	
	High school graduate or equivalent (*n* = 33)	3.6	4.4	2.1	
**Smoking habits (*n* = 898)**		**%**	**%**	**%**	
	No (*n* = 839)	93.4	92.9	94.5	0.202
	Yes (*n* = 21)	2.3	3.0	1.0	
	Occasionally (*n* = 38)	4.2	4.1	4.5	
**How long consulting Instagram (*n* = 794)**		**Mean (SD)**	**Mean (SD)**	**Mean (SD)**	<0.001 ^a^
	Months	23.6 (24.1)	22 (24.2)	26.6 (23.7)	
**Hours per week on Instagram checking for nutrition or exercise (*n* = 879)**		**%**	**%**	**%**	
	Less than 1 h	29. 9	40.4	7.8	<0.001 ^a^
	Between 1–2.5 h	43.3	40.3	49.8	
	Between 2.5–5 h	12.6	10.2	17.7	
	More than 5 h	14.1	9.1	24.7	
**Instagram attitudes (*n* = 898)**					
	Followed a nutritional influencer	%	%	%	
	No (*n* = 505)	56.2	74.1	18.7	<0.001 ^a^
	Yes (*n* = 393)	43.8	25.9	81.3	
	Looked up the influencer’s accreditation	%	%	%	
	No (*n* = 697)	77.6	87.7	56.4	<0.001 ^a^
	Yes (*n* = 201)	22.4	12.3	43.6	
	Checked the accuracy of the recipes	%	%	%	
	No (*n* = 664)	73.9	87.4	45.7	<0.001 ^a^
	Yes (*n* = 234)	26.1	12.6	54.3	
	Hiring or have considered hiring a personal training	%	%	%	
	No (*n* = 732)	81.5	85.2	73.7	<0.001 ^a^
	Yes (*n* = 166)	18.5	14.8	26.3	
**Impact on body image (*n* = 898)**		**%**	**%**	**%**	
	Positive (*n* = 268)	30.0	21.0	48.4	<0.001 ^a^
	Negative (*n* = 149)	16.0	16.1	17.6	
	Neither (*n* = 481)	54.0	62.9	33.9	
**Vigorous physical activity (*n* = 770)**		**%**	**%**	**%**	
	Less than 75 min per week (*n* = 107)	13.9	14.0	13.7	0.899
	More than 75 min per week (*n* = 663)	86.1	86.0	86.3	
**Moderate physical activity (*n* = 751)**		**%**	**%**	**%**	
	Less than 150 min per week (*n* = 355)	47.3	47.8	46.1	0.654
	More than 150 min per week (*n* = 396)	52.7	52.2	53.9	
**Hours of sitting (*n* = 898)**		**%**	**%**	**%**	
	Low risk (*n* = 315)	35.1	33.8	37.7	0.644
	Medium risk (*n* = 411)	45.8	46.1	45.0	
	High risk (*n* = 89)	9.9	10.3	9.0	
	Very high risk (*n* = 83)	9.2	9.7	8.3	

^a^ significant difference between columns. K = $1000.

**Table 2:** Posts by the named nutritional influencers analysis.

Posts by the Named Nutritional Influencers (*n* = 210)	No (%)	Yes (%)
Scientific evidence on post	85.7	14.3
Post promoted a product or supplement	8.6	91.4
Post promoted a brand	6.2	93.8
Post about a recipe	65.7	34.3
Post suggested follower what to eat	13.3	86.7

## Data Availability

The data sets analyzed during the current study are available from the corresponding author on reasonable request. All data analyzed during this study are included in this published article.

## Supplementary Materials

The following supporting information can be downloaded at: https://www.mdpi.com/article/10.3390/foods11020239/s1, Table S1: Sample from which State of residence.

## References

[1] Fernández Bayo I., Menéndez O., Fuertes J. (2019). La Comunidad Científica ante las Redes Sociales. Guía de Actuación Para Divulgar Ciencia a Través de Ellas.

[2] Hajli M.N. (2014). A study of the impact of social media on consumers. Int. J. Mark. Res..

[3] Fung I.C.-H., Blankenship E.B., Ahweyevu J.O., Cooper L.K., Duke C.H., Carswell S.L., Jackson A.M., Jenkins J.C., Duncan E.A., Liang H. (2019). Public Health Implications of Image-Based Social Media: A Systematic Review of Instagram, Pinterest, Tumblr, and Flickr. Perm. J..

[4] Helm J., Jones R.M. (2016). Practice paper of the Academy of Nutrition and Dietetics: Social media and the dietetics practitioner: Opportunities, challenges, and best practices. J. Acad. Nutr. Diet..

[5] Loehmer E., Smith S., McCaffrey J., Davis J. (2018). Examining internet access and social media application use for online nutrition education in SNAP-Ed participants in rural Illinois. J. Nutr. Educ. Behav..

[6] Paige S.R., Stellefson M., Chaney B.H., Chaney D.J., Alber J.M., Chappell C., Barry A.E. (2017). Examining the relationship between online social capital and eHealth literacy: Implications for Instagram use for chronic disease prevention among college students. Am. J. Health Educ..

[7] Malighetti C., Sciara S., Chirico A., Riva G. (2020). Emotional Expression of #body on Instagram. Soc. Media Soc..

[8] Smith A., Anderson M. Social Media Use 2018: Demographics and Statistics. http://www.pewinternet.org/2018/03/01/social-media-use-in-2018/.

[9] Fernandes P.R.M. (2018). Instagram: Investigating the Influence of Healthy Food Posts on Consumer Purchase Intention.

[10] Coates A.E., Hardman C.A., Halford J.C.G., Christiansen P., Boyland E.J. (2019). Food and beverage cues featured in youtube videos of social media influencers popular with children: An exploratory study. Front. Psychol..

[11] Muralidhara S., Paul M.J. (2018). Healthy selfies: Exploration of health topics on Instagram. JMIR Public Health Surveill..

[12] Klassen K.M., Borleis E.S., Brennan L., Reid M., McCaffrey T.A., Lim M.S.C. (2018). What people “like”: Analysis of social media strategies used by food industry brands, lifestyle brands, and health promotion organizations on Facebook and Instagram. J. Med. Internet Res..

[13] Marrugat J., Vila J., Pavesi M., Sanz F. (1998). Estimación del tamaño de la muestra en la investigación clínica y epidemiológica. Med. Clin..

[14] Association W.M. (2013). World Medical Association Declaration of Helsinki: Ethical principles for medical research involving human subjects. JAMA.

[15] Dimock M. Defining Generations: Where Millennials End and Generation Z Begins. https://www.pewresearch.org/fact-tank/2019/01/17/where-millennials-end-and-generation-z-begins/.

[16] Kim Y., Park I., Kang M. (2013). Convergent validity of the international physical activity questionnaire (IPAQ): Meta-analysis. Public Health Nutr..

[17] Owen N., Healy G.N., Matthews C.E., Dunstan D.W. (2010). Too much sitting: The population health science of sedentary behavior. Exerc. Sport Sci. Rev..

[18] Zuckerman M. Estadísticas Globales y Clave Del 2019. https://blog.digimind.com/es/tendencias/instagram-estadísticas-globales-clave-del-2019.

[19] Turner P.G., Lefevre C.E. (2017). Instagram use is linked to increased symptoms of orthorexia nervosa. Eat. Weight Disord. Anorexia, Bulim. Obes..

[20] Fioravanti G., Prostamo A., Casale S. (2020). Taking a Short Break from Instagram: The Effects on Subjective Well-Being. Cyberpsychol. Behav. Soc. Netw..

[21] Alley S., Wellens P., Schoeppe S., de Vries H., Rebar A.L., Short C.E., Duncan M.J., Vandelanotte C. (2017). Impact of increasing social media use on sitting time and body mass index. Health Promot. J. Aust..

[22] About 13.1 Percent Have a Master’s, Professional Degree or Doctorate. https://www.census.gov/library/stories/2019/02/number-of-people-with-masters-and-phd-degrees-double-since-2000.html.

[23] Pilgrim K., Bohnet-Joschko S. (2019). Selling health and happiness how influencers communicate on Instagram about dieting and exercise: Mixed methods research. BMC Public Health.

[24] Hajli N. (2015). Social commerce constructs and consumer’s intention to buy. Int. J. Inf. Manag..

[25] Davis F.D. (1989). Perceived usefulness, perceived ease of use, and user acceptance of information technology. MIS Q..

[26] Sharma S.S., De Choudhury M. Measuring and characterizing nutritional information of food and ingestion content in instagram. Proceedings of the 24th International Conference on World Wide Web.

[27] Brown Z., Tiggemann M. (2016). Attractive celebrity and peer images on Instagram: Effect on women’s mood and body image. Body Image.

[28] Piercy K.L., Troiano R.P., Ballard R.M., Carlson S.A., Fulton J.E., Galuska D.A., George S.M., Olson R.D. (2018). The Physical Activity Guidelines for Americans. JAMA.

