# Supplementary material for: Nutrition-Related Content on Instagram in the United States of America: Analytical Cross-Sectional Study
Source: Foods. 2022 Jan 17;11(2):239. doi: 10.3390/foods11020239 (PMC8774557; doi:10.3390/foods11020239)
Supplement: Supplementary file 1 [file foods-11-00239-s001.zip › foods-1547242-supplementary.pdf]

Supplementary Table S1: Sample from which State of residence.

State	Participants	%
Alabama	10	1.1
Alaska	2	0.2
Arizona	14	1.6
Arkansas	3	0.3
California	59	6.6
Colorado	7	0.8
Connecticut	7	0.8
Delaware	3	0.3
District of Columbia (DC)	5	0.6
Florida	57	6.3
Georgia	23	2.6
Hawaii	1	0.1
Idaho	2	0.2
Illinois	24	2.7
Indiana	15	1.7
Iowa	5	0.6
Kansas	7	0.8
Kentucky	8	0.9
Louisiana	4	0.4
Maine	2	0.2
Maryland	10	1.1
Massachusetts	12	1.3
Michigan	8	0.9
Minnesota	10	1.1
Mississippi	3	0.3
Missouri	13	1.4
Montana	1	0.1
Nebraska	4	0.4
Nevada	3	0.3
New Hampshire	2	0.2
New Jersey	24	2.7
New Mexico	2	0.2
New York	57	6.3
North Carolina	115	12.8
North Dakota	4	0.4
Ohio	20	2.2
Oklahoma	9	1.0
Oregon	7	0.8
Pennsylvania	22	2.4
Rhode Island	1	0.1
South Carolina	12	1.3
South Dakota	1	0.1
Tennessee	10	1.1

Texas	238	26.5
Utah	5	0.6
Vermont	1	0.1
Virginia	17	1.9
Washington	17	1.9
Wisconsin	11	1.2
Wyoming	1	0.1
Total	898	100.0
